# Nonlinear Elasticity and Damage Prediction in Automated Fiber Placement Composites via Nested Micromechanics

**DOI:** 10.3390/ma18143394

**Published:** 2025-07-19

**Authors:** Hadas Hochster, Gal Raanan, Eyal Tiosano, Yoav Harari, Golan Michaeli, Yonatan Rotbaum, Rami Haj-Ali

**Affiliations:** School of Mechanical Engineering, Tel Aviv University, Tel Aviv 6997801, Israel; hochster@mail.tau.ac.il (H.H.); galraanan@mail.tau.ac.il (G.R.); yoavharari2@gmail.com (Y.H.); golanm@mail.tau.ac.il (G.M.); y.rotbaum@gmail.com (Y.R.)

**Keywords:** composite materials, automated fiber placement (AFP), micromechanical modeling

## Abstract

Automated fiber placement (AFP) composites exhibit complex mechanical behaviors due to manufacturing-induced mesostructural variations, including resin-rich regions and tow gaps that significantly influence both local stress distributions and global material responses. This study presents a hierarchically nested modeling framework based on the Parametric High-Fidelity Generalized Method of Cells (PHFGMC) to predict the effective elastic properties and nonlinear mechanical response of AFP composites. The PHFGMC model integrates micro- and meso-scale analyses using representative volume elements (RVEs) derived from micrographs of AFP composite laminates to capture these manufacturing-induced characteristics. Multiple RVE configurations with varied gap patterns are analyzed to quantify the influence of mesostructural features on global stress–strain response. Predictions for linear and nonlinear elastic behaviors are validated against experimental results from carbon fiber/epoxy AFP specimens, demonstrating good quantitative agreement with measured responses. A cohesive extension of the PHFGMC framework further captures damage initiation and crack propagation under transverse tensile loading, revealing failure mechanisms specifically associated with tow gaps and resin-rich areas. By systematically accounting for manufacturing-induced variability through detailed RVE modeling, the nested PHFGMC framework enables the accurate prediction of global mechanical performance and localized behavior, providing a robust computational tool for optimizing AFP composite design in aerospace and other high-performance applications.

## 1. Introduction

Fiber-reinforced polymer (FRP) composites are widely used in the aviation industry for their lightweight and high-strength properties, significantly enhancing fuel efficiency and aircraft performance [1,2,3,4]. However, their conventional slow manufacturing process often involves high costs in terms of labor and materials as well as complex production processes, which can lead to variability in mechanical strength and quality. Advanced manufacturing techniques, such as automated fiber placement (AFP), have been developed to address some of these challenges [5].

AFP is an advanced method of manufacturing composite materials that employs gantry and robotic articulated arm systems to lay down composite tows onto designated surfaces precisely, controlling fiber orientation and placement with high accuracy [6,7]. This technique offers notable advantages, including exceptional precision in fiber placement, minimized material waste, and the capability to produce complex geometries with consistent quality, making it highly suitable for high-performance sectors such as aerospace and automotive [8]. Despite its advantages, AFP composites may contain many defects that can occur during the manufacturing process, e.g., excessive gaps and overlaps of fiber tows due to misalignment [9,10,11], as well as wrinkling [12,13], twisting [14], tow-drops [15], waviness and bunching of tows [12,16]. These defects may significantly affect the uniformity of the composite’s mesostructure, impacting its mechanical properties and overall performance [17,18], highlighting the need for precise process controls and advanced research to ensure the consistent, high-quality production of AFP composites.

Ensuring consistent quality in AFP composites is challenging due to their sensitivity to the AFP manufacturing parameters that affect both microstructure and mesostructure. Variations in calibration settings during the AFP program can significantly influence features such as fiber volume fraction, resin-rich interlaminar areas, and void content, which directly impact the composite’s mechanical properties and performance [19,20,21,22,23,24]. Even with precise AFP processes, gaps between tows may still occur due to slight tolerances in fiber placement, leading to localized differences in fiber volume fraction and resin-rich areas. As observed in micrographs, these gaps can vary in length and shift between plies [25]. Despite the technological precision of AFP, such variations can disrupt the uniformity of the composite’s mesostructure [26], causing uneven load distribution or leading to the development of defect-induced failure mechanisms. This highlights the need for a comprehensive mechanical analysis to understand these issues better and address them.

Recent research highlights the critical role of advanced micromechanical and mesomechanical modeling approaches in addressing the complexities arising from manufacturing variations in AFP composites. The study conducted by Le Reun et al. [27] introduced a microscale thermomechanical model for laser-assisted AFP of thermoplastic composites aimed at improving predictions of temperature distribution and in situ consolidation. It incorporates the thermal properties of the fibers and matrix, along with factors such as laser incidence angle and fiber distribution. The model was validated against homogenized models under various heating conditions. In the field of meso-macro mechanical failure, Zhou et al. [28] studied how gaps affect the failure mechanisms of AFP-manufactured laminates under out-of-plane tensile loading. Using micrographs of the laminate’s internal mesostructure, a numerical model was developed to predict how resin-rich areas surrounding gaps influence structural performance and damage patterns. In addition, these micrographs have provided insights into the internal structure, enabling a detailed comparison of the effects of AFP in situ consolidation and autoclave reconsolidation. Using micrographs, Pourahmadi et al. [28] developed a two-dimensional representative volume element (RVE) to predict specific mechanical elastic properties of thermoplastic composites. These micrographs provided insight into the internal structure, enabling a detailed comparison of the effects of in situ AFP consolidation and autoclave reconsolidation. The images revealed that autoclave reconsolidation significantly reduced void content, resulting in enhanced mechanical properties compared to AFP in situ consolidation. In another micromechanical study, the work by Ghayour et al. [29] presented a meso-macro numerical model to examine the effect of gaps on the damage behavior of AFP composites. Their approach employed the Generalized Method of Cells (GMC) micromechanical model [30] to homogenize the resin-gap microstructure between tows using RVEs of varying gap sizes, which was then integrated into a macro-scale model based on the Continuum Damage Mechanics (CDM) framework.

However, it remains challenging to capture the nonlinear behavior of AFP composites, considering their unique mechanical signature. The Parametric High-Fidelity Generalized Method of Cells (PHFGMC) [31] is applied to evaluate the effect of the microstructure and mesostructure on the nonlinear behavior of AFP composites. Micromechanical modeling approaches, including PHFGMC and other RVE-based frameworks such as classical GMC, finite element RVE techniques, and homogenization-based methods [32,33,34], are widely employed to describe the influence of microstructural architecture on the effective mechanical behavior of composite materials. To address this, the PHFGMC is an advanced micromechanical approach well-suited for analyzing the nonlinear and failure behavior of diverse periodic composite materials [35,36,37,38]. Its primary goal is to accurately predict the effective mechanical properties and the local elastic fields within composites with periodic microstructures. This micromechanical method is highly effective in predicting the overall nonlinear behavior and damage responses of heterogeneous multiphase composites. The PHFGMC method imposes periodicity conditions on the boundaries of an RVE, which can be divided into general quadratic or hexahedral subcells representing the fiber and matrix phases. Continuities in average traction and displacement are enforced between subcells to maintain equilibrium. Additionally, a recent formulation of the PHFGMC, based on average virtual work, generates a symmetric stiffness matrix that is solved through a nonlinear iterative process. This formulation enhances computational efficiency and is well-suited for micromechanical calculations compared to traditional Finite Element (FE) methods.

This study combines experimental and numerical approaches to investigate AFP composites. The PHFGMC micromechanical model is proposed to predict the effective global properties and nonlinear mechanical responses of AFP composites while accounting for variations in the mesostructure. The analysis employs a nested approach, accounting for the tow material’s microstructure and the AFP composites’ mesostructure as observed in micrographs. Additionally, different RVEs are analyzed to examine how various mesostructures influence the global response of the composite laminate. The study includes coupon testing to evaluate the PHFGMC-AFP model’s capability to predict the nonlinear mechanical behavior of AFP composites.

The first section introduces the materials and methods, the governing equations of the PHFGMC, and the materials and testing standards used. This section outlines the procedures for preparing micrographic specimens and the observed microstructure and mesostructure. The results and discussion section follows, presenting hierarchically nested analyses based on micro-level repeating unit cell (RUC) and meso-level RVE developed from micrographic data. This section also provides the input material properties for each phase and the laminate’s effective properties as predicted by the PHFGMC. Subsequently, experimental results and PHFGMC numerical analyses are included to predict the stress–strain responses of the AFP laminate. Next, by incorporating cohesive PHFGMC subcells within the RVE, the model accurately simulates damage progression, including matrix cracks and delaminations, under transverse-tensile loading. This approach allows detailed prediction of the composite’s stress–strain response and highlights the critical role of mesostructural features in governing failure mechanisms. In the next section, an analysis comparing different RVEs is provided to evaluate the global mechanical behavior of the laminate, verifying the accuracy of the nested PHFGMC approach in predicting the global effective response of AFP composites. Finally, the conclusions of the results are presented.

## 2. Materials and Methods

### 2.1. The PHFGMC Micromechanical Model

In this study, the PHFGMC is applied to predict the nonlinear response of AFP composites, taking into account their micromechanical and mesomechanical characteristics. The formulation is also based on the average virtual work principle, which improves numerical efficiency and stability. A detailed formulation of the PHFGMC method is provided in [31].

**Figure 1:**
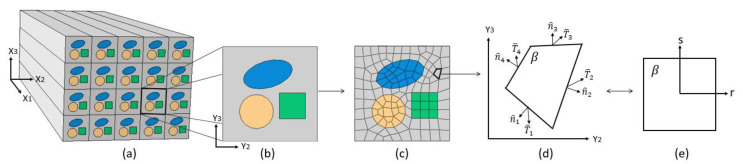
Schematic representation of (**a**) a doubly periodic material at composite level, (**b**) the RVE used to model the composite’s microstructure, and (**c**) the RVE divided into arbitrary quadrilateral subcells. The mapping of subcell *β* from the physical coordinate system to the parametric coordinate system is shown in (**d**,**e**).

In the PHFGMC framework, the microstructure of the material system is identified within an RVE. Next, the RVE is divided into hexahedral-shaped subcells, with each subcell representing either the matrix or fiber phase. The hexahedral shape of the subcells efficiently represents the complex microstructure and geometry with a minimal number of subcells, as illustrated in Figure 1. This method defines the composite material using a global coordinate system $\left(x_{1},x_{2},x_{3}\right)$, while the identified RVE is described in a local coordinate system $\left(y_{1},y_{2},y_{3}\right)$. Subsequently, each subcell is mapped to the parametric coordinate system $\left(r,s,t\right)$.

The following displacement expansion for subcell β, in terms of the microvariables and the parametric coordinate system is given by

(1) $$u^{\left(\beta \right)}=\varepsilon_{0}\cdot x+W_{0}^{\left(\beta \right)}+\frac{1}{2}(W_{2}^{\left(\beta \right)}-W_{4}^{\left(\beta \right)})r+\frac{1}{2}(W_{3}^{\left(\beta \right)}-W_{1}^{\left(\beta \right)})s\;+\frac{1}{4}(W_{2}^{\left(\beta \right)}+W_{4}^{\left(\beta \right)}-2W_{0}^{\left(\beta \right)})(3r^{2}+rs-1)\;+\frac{1}{4}(W_{1}^{\left(\beta \right)}+W_{3}^{\left(\beta \right)}-2W_{0}^{\left(\beta \right)})(3s^{2}+rs-1)$$

where $\varepsilon_{0}\cdot x$ is the applied remote global displacement field, including the remote strain field applied on the composite, $W_{0}^{\left(\beta \right)}$ is a center microvariable, and the microvariables $W_{i}^{\left(\beta \right)}$(i=1,2,3,4) represent the average displacement of the *i*th subcell face.

Average virtual work is defined locally using subcell face-average virtual displacement microvariables related to average face tractions. At the global level, this average virtual work is expressed in terms of remote virtual strains and their corresponding average stresses. The balance of external and internal average virtual work (δ) for subcell β is given as follows [36]:

(2) $${\int_{V}\delta \varepsilon }^{(\beta ),T}\sigma^{\left(\beta \right)}dV=\delta \varepsilon_{0}^{(\beta ),T}{f_{U}}^{\left(\beta \right)}+\delta W^{(\beta ),T}f_{W}^{\left(\beta \right)}$$

where $\varepsilon^{\left(\beta \right)}$ is the strain vector described by the microvariables:

(3) $$\varepsilon^{\left(\beta \right)}=\varepsilon_{0}+A_{W}^{\left(\beta \right)}W^{\left(\beta \right)}$$

where the coefficient matrix ${A_{W}}^{\left(\beta \right)}$ relates to the subcell geometry, including Jacobian components. Hooke’s law describes the linear stress–strain relation for a material within a subcell:

(4) $$\sigma^{\left(\beta \right)}=C^{\left(\beta \right)}\varepsilon^{\left(\beta \right)}=C^{\left(\beta \right)}\left(\varepsilon_{0}+A_{W}^{\left(\beta \right)}W^{\left(\beta \right)}\right)$$

where $\sigma^{\left(\beta \right)}$ is the Cauchy stress tensor, and $C^{\left(\beta \right)}$ is the stiffness matrix of the material.

Next, $f_{U}$ and $f_{W}$ represent the subcell’s internal resisting vectors corresponding to the global displacement and the microvariables, respectively. These vectors can be expressed as follows:

(5) $${f_{U}}^{\left(\beta \right)}=\int_{V}\sigma^{\left(\beta \right)}dV,\;f_{W}^{\left(\beta \right)}=\int_{V}A_{W}^{(\beta ),T}\sigma^{\left(\beta \right)}dV$$

Substituting Equation (4) into Equation (5) gives the following result:

(6) $${f_{U}}^{\left(\beta \right)}=\int_{V}C^{\left(\beta \right)}\left(\varepsilon_{0}+A_{W}^{\left(\beta \right)}W^{\left(\beta \right)}\right)dV,\;\;f_{W}^{\left(\beta \right)}=\int_{V}A_{W}^{(\beta ),T}C^{\left(\beta \right)}\left(\varepsilon_{0}+A_{W}^{\left(\beta \right)}W^{\left(\beta \right)}\right)dV$$

The equilibrium residual for the complete system of equations is generally expressed as follows:

(7) $$R\equiv f-P=\left\{\begin{array}{c} \int_{V}A_{W}^{(\beta ),T}\sigma^{\left(\beta \right)}dV\\ \int_{V}\sigma^{\left(\beta \right)}dV \end{array}\right\}-\left\{\begin{array}{c} P_{W}\\ P_{\varepsilon_{0}} \end{array}\right\}=\left\{0\right\}$$

where f is the internal force vector, and P is the external force vector; $P_{W}$ is the external force vector, nonzero for free internal surfaces under pressure loading, and $P_{\varepsilon_{0}}$ is a generalized force in macroscale terms.

Following that, the incremental equation equilibrium of Equation (2) can be expressed by

(8) K u^=∫VAWTCAWdV∫VAWTCdV∫VCAWdV∫VCdV(t)Wε0(t+Δt)=R(t+Δt)

By assembling the contributions of the RVE subcells to the global system, R represents the overall generalized force vector, $\;\hat{u}=\left(W,\varepsilon_{0}\right)$ are the RVE displacement variables, and K is the symmetric stiffness matrix.

Accordingly, the effective stiffness matrix $C^{*}$ can be determined by summing the contributions of each subcell:

(9) $$C^{*}=\frac{1}{S_{total}}\sum_{\beta =1}^{N_{sc}}S_{\beta }C^{\left(\beta \right)}G^{\left(\beta \right)}$$

with $S_{total}$ as the total area of the RVE, $N_{sc}$ the number of subcells in the RVE, and $G^{\left(\beta \right)}$ is the strain concentration tensor. This strain concentration tensor relates the external remote strain $\varepsilon_{0}$ applied to the composite to the corresponding local average strain within the subcell ${\;\bar{\varepsilon }}^{\left(\beta \right)}$:

(10) $${\;\bar{\varepsilon }}^{\left(\beta \right)}=\frac{1}{S_{\beta }}\int_{S_{\beta }}\varepsilon \left(y\right)dS\equiv G^{\left(\beta \right)}:\varepsilon_{0}$$

As a result, the calculated effective (homogenized) stiffness matrix connects the average global stress of the composite to the average strain:

(11) $$\;\bar{\sigma }=C^{*}\;\bar{\varepsilon }$$

### 2.2. Material System and Coupon Testing

To investigate the influence of the AFP manufacturing process on thermoset FRP composites, coupon and cross-section micrograph specimens were extracted from AFP-fabricated plates. The material used in this study is M21/34%/UD194/IMA-12K, a commercially available prepreg consisting of intermediate modulus carbon fiber combined with the M21 epoxy matrix system. According to the manufacturer’s datasheet, the fiber volume fraction is approximately 60% [39].

Fabrication was performed using a robotic arm equipped with an AFP modular head, which carried eight spools of 6.35 mm (0.25″) wide tows. Each course consisted of eight tows, resulting in a total course width of 50.8 mm (2″). The gap between adjacent courses was set according to aerospace standards, which include a built-in offset between neighboring laminae. A 21-ply unidirectional laminate was produced with an average total thickness of 3.95 mm.

Nominal pressure was applied during the layup process to achieve sufficient compaction of the laminate stackup. This pressure, combined with a strict bagging procedure, ensured the quality of the cured laminate. The roller is manufactured and designed in order to provide complete coverage of the tow courses in the designated sequence. Following the AFP process, the composite laminate was cured in an autoclave to complete the curing process and full polymerization of the material. The cure schedule followed the manufacturer’s recommended autoclave process and was carefully executed during laminate fabrication. All process conditions and requirements were met, and no variations in the parameters were observed.

**Figure 2:**
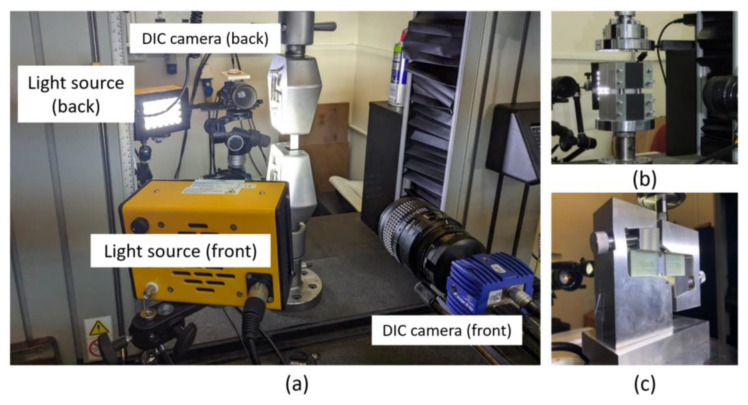
Test setups: (**a**) tensile, (**b**) compression, and (**c**) shear.

Coupons and micrograph specimens were precisely cut from the AFP fabricated plate using a water jet cutting machine, minimizing the risk of delamination. Coupon tests, including tensile (axial and transverse), compression (axial and transverse), and in-plane shear tests, were conducted according to ASTM D3039 [40], ASTM D6641 [41], and ASTM D5379 [42] standards. To ensure consistency, specimens were cut from the same laminate plate and along the same line, preserving the designed gap pattern across all samples of the same test. This approach ensured comparable mesostructural characteristics across specimen types, enabling a reliable comparison of their mechanical responses. The dimensions of the specimens are summarized in Table 1. A speckled pattern, created by spraying a random pattern of black and white, was applied within the test region to enable 2D digital image correlation (DIC) analysis. The tests were performed using an Instron 5582 testing machine equipped with a 100 kN load cell and operated at a constant displacement rate of 0.3 mm/min. Two LaVision Imager M-lite cameras (LaVision GmbH, Göttingen, Germany) and light sources positioned to illuminate the specimens were placed in front of and behind the specimens to capture data for DIC analysis using the LaVision Davis 10.1.2 software. Images were taken at 1 s intervals throughout the test duration, and the cameras were synchronized with the test system. DIC analysis was performed to obtain a full-field strain map over the entire gauge section. The test setups are shown in Figure 2.

### 2.3. Micrographs of AFP Specimens

The numerical analysis began with optical micrographs of the material samples, which were extracted to ensure that the captured surface represented the material’s thickness, allowing for visualization of the fibers in cross-section. The specimens were embedded in liquid resin, cured, and then polished with varying grit paper to achieve a smooth, flat surface, enabling precise observation of the internal structure under a microscope. An Olympus LEXT OLS4100 confocal microscope (Olympus Corporation, Tokyo, Japan) was used to capture images of both the microstructure and mesostructure at magnifications ranging from 5× to 50×.

In this study, “mesostructure” refers to the micrograph of the laminate through its thickness, while “microstructure” describes the arrangement of fibers and matrix within a single lamina tow. The imaging of the laminate through its thickness enabled the observation of AFP’s mechanical signature, including variations in tow gaps, fiber misalignment, and resin-rich regions. A closer examination of the lamina’s microstructure was carried out by increasing the microscope’s magnification. These micrographs focused on the tows, with gaps between adjacent tows being analyzed in the mesostructure.

Observing the microstructure of FRP composites is essential for analyzing fiber alignment and determining the fiber volume fraction. Once these parameters are established, a homogenization procedure can be employed to assess the composite material’s mechanical response. Additionally, studying the microstructure under specific loading conditions provides valuable insights into how the constituent materials contribute to the material’s overall behavior. This approach is also effective for detecting voids and other defects to ensure the integrity of the manufacturing process.

## 3. Results and Discussion

### 3.1. Experimental Results

The experimental results include axial tension, axial compression, transverse tension, transverse compression, and in-plane shear tests, based on the five most representative experiments. These are compared with the stress–strain responses predicted by the PHFGMC within the nested micromechanical–mesomechanical framework. The corresponding experimental stiffness values for each loading condition are summarized in Table 2.

The axial tensile test results indicated a longitudinal modulus ranging from 145.6 to 165.0 GPa, with an average of 155.6 GPa and a standard deviation of 4.7%. The Poisson ratio was obtained as 0.32, with a standard deviation of 2.7%. The AFP process and the presence of gaps may contribute to a reduction in longitudinal modulus, as evidenced by the variability in results, with values lower than those of hand-layup manufactured specimens. Similar trends have been noted in the literature for AFP composites [25], where the gaps between courses can introduce shear effects that influence the failure stress, particularly under tensile loading. The average longitudinal compressive modulus was 142.2 GPa, with a standard deviation of 4.1%. These results suggest that the AFP process and the presence of gaps have a limited effect on compressive stiffness.

The results of the transverse tensile and compression tests showed noticeable variation in the slope of the curves between specimens, indicating differences in Young’s modulus. In the tensile tests, Young’s modulus ranged from 8.10 GPa to 10.3 GPa, with an average of 9.07 GPa and a standard deviation of approximately 12%. In the compression tests, Young’s modulus ranged from 7.71 GPa to 11.1 GPa, with an average of 9.42 GPa and a standard deviation of approximately 18%. Both the transverse tension and compression results exhibit a wider distribution and higher variability than those observed in the axial and shear cases. This scatter is expected, as transverse behavior in unidirectional composites is matrix-dominated and thus highly sensitive to local mesostructural defects such as tow gaps, resin-rich regions, and interfacial irregularities—features that are particularly prevalent in AFP-manufactured laminates.

Subsequently, in the shear test results, the nonlinear shear response is visible and effectively captured by the PHFGMC model. The calculated average engineering shear modulus was 5.41 GPa, with a standard deviation of 3.3%. Notably, the shear response demonstrates minimal sensitivity to the mechanical signature of the AFP composite, aligning with expectations for traditional composite laminates.

### 3.2. Nested PHFGMC for the Nonlinear Analysis of AFP Composites

**Figure 3:**
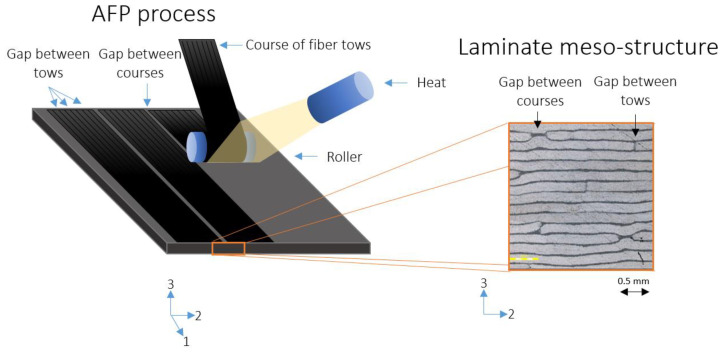
An illustration of the AFP manufacturing process and a micrograph of the mesostructure. The micrograph shows the cross-section of the unidirectional laminate, with dark regions representing resin-rich areas, highlighting the interfaces between plies and the gaps between courses and tows. The light gray areas represent the fiber tows.

The initial microscopy inspection of the specimens revealed the fiber-rich regions within the plies, while the interlaminar interface consisted only of the matrix phase without fibers. Figure 3 illustrates an example of the mesostructure observed in the micrograph, showing fibers aligned along the 1-axis direction. The resin-rich interlaminar interface was measured to be 30 µm wide. This finding is unique to the AFP manufacturing technique. In contrast, in a hand layup unidirectional laminate, the resin-rich regions between the plies are typically less distinct, with a distribution of fibers throughout the interlaminar area [43,44]. The interlaminar interface region observed in the AFP composite has been highlighted in previous studies [45,46]. This may be influenced by factors such as the compaction force applied by the rollers during the AFP process, the temperature at the roller tip, the autoclave curing process, and other related parameters [20,25].

Similarly, the gaps between adjacent tows within a single course, identified as intralaminar resin-rich areas, were measured at 20 µm wide. Additionally, the gap between the two adjacent courses was measured to be 0.5 mm. Both types of gaps contribute to the formation of intralaminar resin-rich areas, which can lead to localized stress distributions. Although these gaps can be managed by adjusting AFP parameters, even minimal gap sizes can generate a repeating pattern of epoxy lines through the thickness of the lamina, occurring between each pair of tows and courses. This phenomenon can be critical, potentially leading to microcracking and damage propagation under various mechanical loads [47].

**Figure 4:**
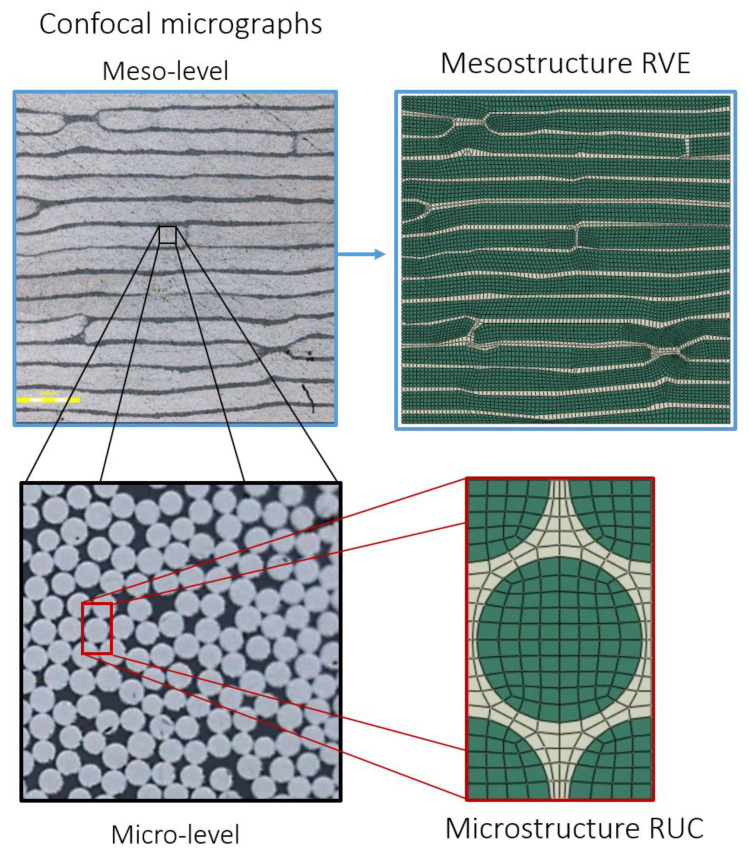
The meso-level micrograph captures the full-thickness cross-section of the unidirectional laminate, while the micro-level micrograph zooms in and focuses on the arrangement of fibers and matrix within an individual tow of the lamina. The microstructure is modeled by an idealized hexagonal array RUC, whereas the mesostructured RVE directly represents the mesostructure.

Figure 4 shows micrographs of the mesostructure and microstructure used to establish the RVEs defining the AFP composite at the meso and micro levels. As previously mentioned, the “mesostructure” refers to the cross-sectional micrograph of the laminate through its entire thickness, while the “microstructure” illustrates the arrangement of fibers and matrix within an individual lamina. The RVEs were generated and meshed in ABAQUS 2022 finite element software before being analyzed using the PHFGMC approach. The PHFGMC nested approach presented in this study focuses on predicting the effective properties of the tow using the microstructure RUC based on the calibrated properties of the IMA fiber and M21 matrix. These predicted tow properties are then applied in the PHFGMC mesostructure analysis, along with the M21 matrix properties, to estimate the effective properties of the AFP laminate.

The microstructure of the AFP laminate, as observed in the micrographs, closely resembled the expected characteristics of unidirectional hand layup laminates, with fibers arranged in a random pattern across the cross-section. The process involved analyzing 45 micrographs to calculate the fiber area as a percentage of the total image area. Based on this analysis, the average fiber volume fraction was found to be 76%, with a standard deviation of 2.3%. Therefore, an idealized hexagonal-packed RUC was chosen to represent the tow microstructure and was labeled as the “microstructure RUC.” Although the observed random fiber arrangement in the microstructure could be modeled directly, choosing an idealized hexagonal array RUC simplifies the calculations, improves computational efficiency, and offers a reliable approximation, which is crucial for practical simulations. The inputs for the microstructure RUC included the fiber and matrix properties, while the PHFGMC numerical simulations resulted in the effective properties of the tow, corresponding to the fiber-rich regions within the lamina. The IMA fiber was modeled as a linear orthotropic material, with its elastic properties summarized in Table 3. The M21 matrix was defined as a linear isotropic material, and its mechanical properties are provided in Table 4. The fiber and matrix properties were initially calibrated using values from the manufacturer’s datasheet [39] and supplemented with literature sources [48]. The effective elastic properties of the tow, predicted using the PHFGMC based on the microstructural RUC model, are presented in Table 5.

The next step of the PHFGMC nested approach is to predict the laminate’s effective properties and nonlinear stress–strain response. This is achieved by simulating the “mesostructure RVE,” which captures the mechanical signature of the AFP composite, including resin-rich interfaces between the plies, gaps between courses, and gaps between tows. This detailed representation enables accurate prediction of the mechanical response accordingly. The inputs for the mesostructure RVE include the tow’s effective properties, as predicted by the micromechanical PHFGMC analysis, and the M21 matrix properties. The matrix properties used in the mesostructure PHFGMC analysis are similar to those used in the microstructure analysis based on the assumption that the matrix within the tows behaves similarly to the matrix in the gaps. The nonlinear constants for both the tow and matrix were calibrated to fit the Ramberg–Osgood formulation, as presented in Equation (12). The tow is modeled as an orthotropic material, with its linear mechanical properties and calibrated Ramberg–Osgood constants provided in Table 5 and Table 6, respectively. The linear mechanical properties and nonlinear constants of the M21 matrix are shown in Table 7. The Ramberg–Osgood (RO) equation represents the nonlinear plastic deformation [49]:

(12) $$E\varepsilon =\sigma +\alpha {\left(\frac{\left|\sigma \right|}{\sigma_{0}}\right)}^{n-1}\sigma$$

where σ is the stress, ε is the strain and E is Young’s modulus. $\sigma_{0},\alpha$ and n are material parameters defining the nonlinear response: $\sigma_{0}$ is the yield or reference stress, α is an offset coefficient, and n is the strain hardening exponent.

Thus far, the PHFGMC-AFP has been calibrated and verified against linear and nonlinear test data with no post-ultimate damage and elastic degradations. Progressive damage is considered beyond the scope of the current study. Next, we demonstrate how the refined multiscale modeling framework can be used to investigate AFP manufacturing parameters and variations at the meso-scale. Four diverse mesostructure RVEs with different gap patterns were analyzed using the PHFGMC model to examine this influence. In the PHFGMC approach, the RVE represents the smallest volume that reflects the composite’s laminate mesostructure, with periodic boundary conditions applied along its edges to reflect the response of the entire lamina. Employing RVEs with varied gap patterns introduced by the AFP process enables the study of how mesostructural differences affect the material. The periodic boundary conditions ensure that the mechanical properties derived from the RVEs reflect the overall mechanical behavior expected throughout the composite lamina.

The microstructure RUC is the basis for determining the effective properties of the tows (as shown in Table 5) within each mesostructure RVE, even as the gap patterns between the mesostructure RVEs vary. This integration of consistent micromechanical properties across various mesostructure configurations is a core principle of the nested-PHFGMC approach. This is based on the assumption that variations in gap patterns influence only the mesostructure of the laminate, leaving the mechanical properties of the tow—determined through the micromechanical PHFGMC analysis—unchanged.

**Figure 5:**
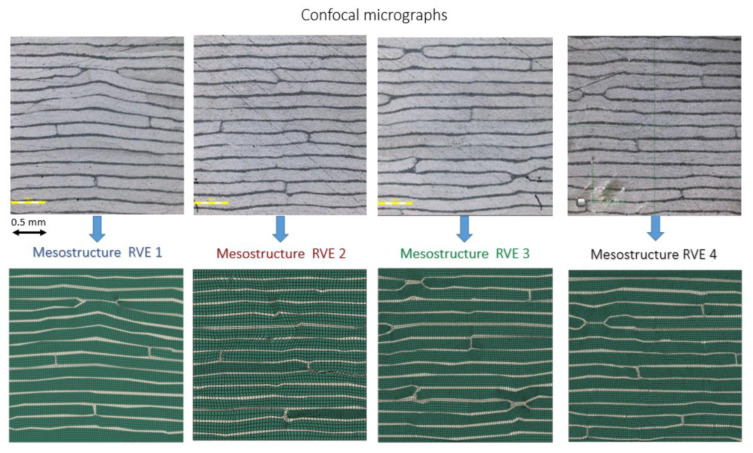
Laminate micrographs of diverse gap patterns between the tows and courses highlight the mesostructure variability. These micrographs serve as the basis for generating mesostructure PHFGMC-RVEs, enabling a detailed analysis of the composite behavior.

**Figure 6:**
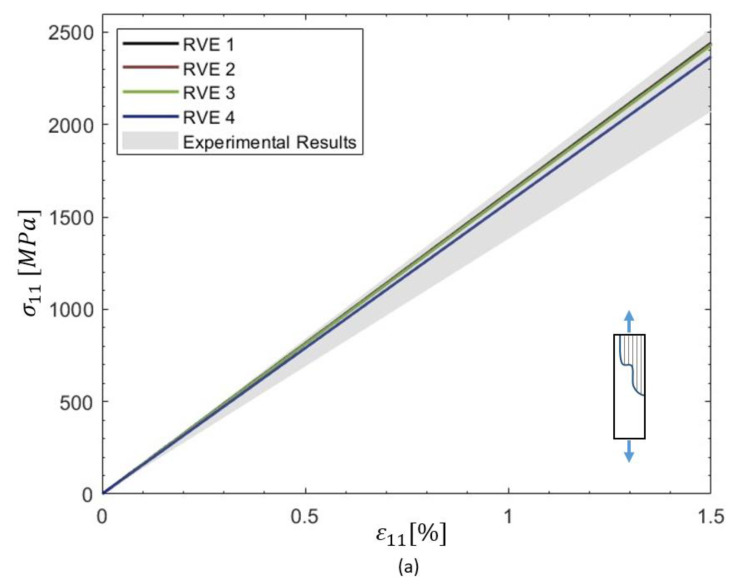

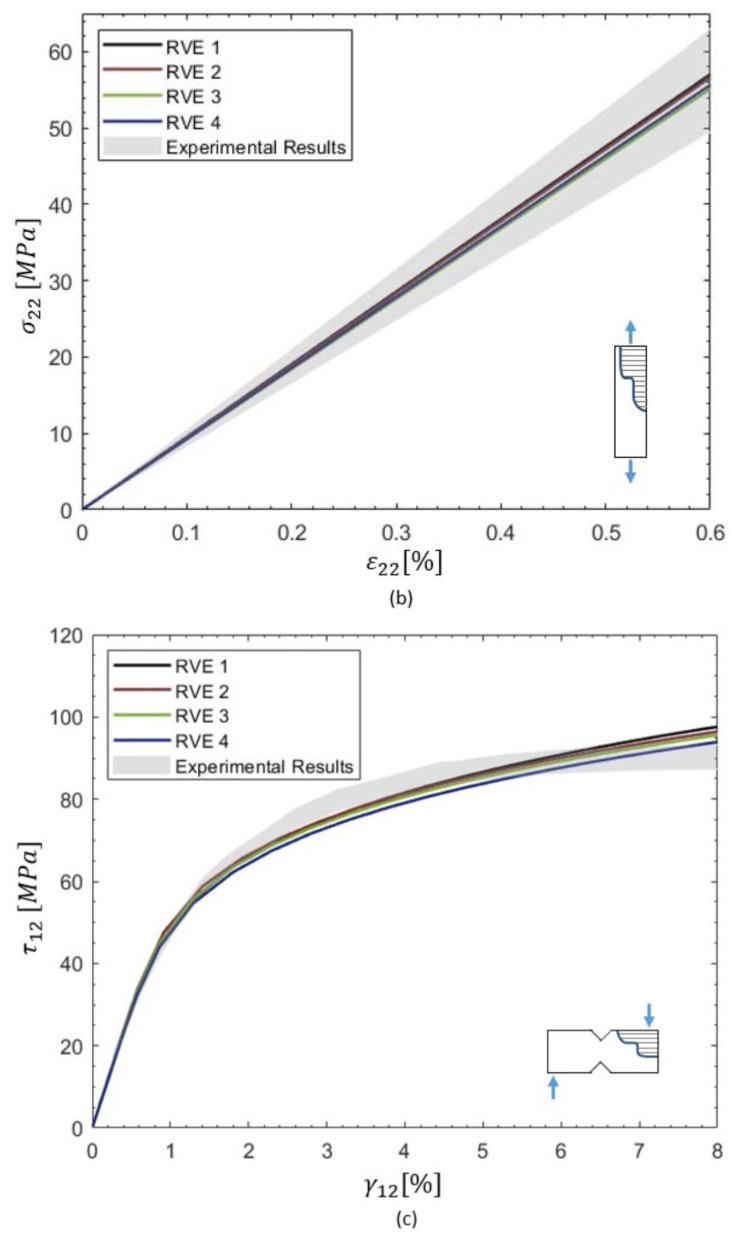
Predicted stress–strain responses for these RVEs under different loading conditions, (**a**) axial tension, (**b**) transverse tension, and (**c**) shear for different gap pattern mesostructure RVEs.

Figure 5 presents mesostructure micrographs showing various gap patterns between tows and courses, which served as references for generating the PHFGMC-RVEs. Each RVE represents a laminate cross-section, capturing the unique gap patterns characteristic of the structure. The stress–strain responses predicted by the PHFGMC for various loading conditions, including axial tension, transverse tension, and shear, are presented in Figure 6. The variability among the different analysis results is quantified by a standard deviation of 1.3% for axial tension, 0.7% for transverse tension, and 1.5% for shear. The PHFGMC results demonstrate that variations among mesostructure RVEs have only a minor influence on the predicted global effective properties and the nonlinear mechanical response of the AFP laminate, even under diverse loading conditions. This significant result highlights the consistency and reliability of AFP composites despite the unique mechanical signature introduced during the manufacturing process.

As a result, the effective global properties of the AFP composite, predicted by the PHFGMC nested approach, are presented in Table 8. This table compares the predicted effective properties with the mechanical properties obtained from the experimental study. The experimental results represent the average mechanical properties of the AFP composite, derived from the stress–strain responses of the axial tensile, transverse tensile, and shear specimens. As shown, the PHFGMC results demonstrate the model’s ability to predict all linear effective mechanical properties of the composite with good accuracy.

The PHFGMC model predicts a longitudinal tensile modulus of 161 GPa, which is 3.2% higher than the experimental average of 156 GPa and 2.4% lower than the experimental maximum of 165 GPa. This close agreement suggests that the model effectively captures longitudinal stiffness, with the slight overestimation likely attributed to idealized fiber alignment or limited representation of manufacturing-induced imperfections. For axial shear, the PHFGMC predicts a shear modulus that is 9.4% higher than the experimental value of 5.41 GPa. The consistency of the experimental shear modulus reflects typical composite behavior and suggests that AFP-specific features have a limited influence on in-plane shear stiffness. Regarding transverse tensile stiffness, the model predicts a modulus of 9.18 GPa, closely matching the experimental average of 9.07 GPa and yielding a relative error of just 1.3%. This strong correlation highlights the model’s capability to capture transverse stiffness accurately, even in the presence of mesostructural features such as resin-rich regions and tow gaps, which strongly influence the mechanical response.

### 3.3. Crack Propagation in Transverse-Tensile Loading

To investigate the influence of mesostructure on crack propagation, the Cohesive Parametric High-Fidelity Generalized Method of Cells (cohesive PHFGMC) [36] model is employed. This advanced micromechanical framework is specifically developed to analyze the behavior of multiphase composite materials, with a focus on damage initiation, evolution, and crack propagation. The cohesive PHFGMC extends the traditional PHFGMC by incorporating a local cohesive zone formulation within the RVE, enabling the simulation of discontinuities such as matrix cracks and delaminations. It employs the traction–separation law defined by the Park–Paulino–Roesler (PPR) [50] model to capture the nonlinear interface behavior effectively. A key advantage of the cohesive PHFGMC approach is its ability to assign cohesive behavior to all RVE subcells and interfaces, allowing for the detailed prediction of damage progression along various paths. This capability supports the reliable evaluation of the composite’s stress–strain response under complex loading conditions.

**Figure 7:**
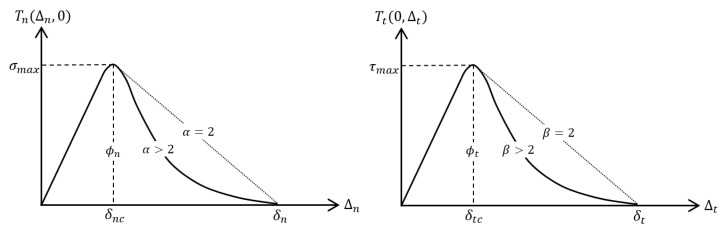
Schematic representation of normal and tangential cohesive parameters defined by PPR model [50] and used in cohesive PHFGMC approach [36].

To implement this approach, RVE1 was meshed with cohesive subcells throughout the matrix and fiber regions. The subcells were divided into tow and matrix regions, each assigned unique properties to reflect the heterogeneous characteristics of the composite mesostructure. This meshing strategy allows the model to account for mesostructural variations and provides a basis for simulating the initiation and propagation of cracks within the composite microstructure. The cohesive behavior in the model is characterized by parameters that define the traction–separation response, including the maximum stress and corresponding fracture energy. Additional parameters specify the shape of the traction–separation curve (α and β), and the initial stiffness components in the normal ($\lambda_{n}=\delta_{nc}/\delta_{n}$) and shear ($\lambda_{t}=\delta_{tc}/\delta_{t}$) directions. These parameters are illustrated in Figure 7. The fracture energy values implemented in the model were derived from experimental results of Double Cantilever Beam (DCB) and End-Notched Flexure (ENF) tests [51], ensuring that the cohesive law accurately represents the composite’s fracture behavior; these tests were performed on materials with comparable properties, providing a reasonable approximation for the AFP laminate. A complete summary of the cohesive properties assigned to the tow and matrix subcells is provided in Table 9.

**Figure 8:**
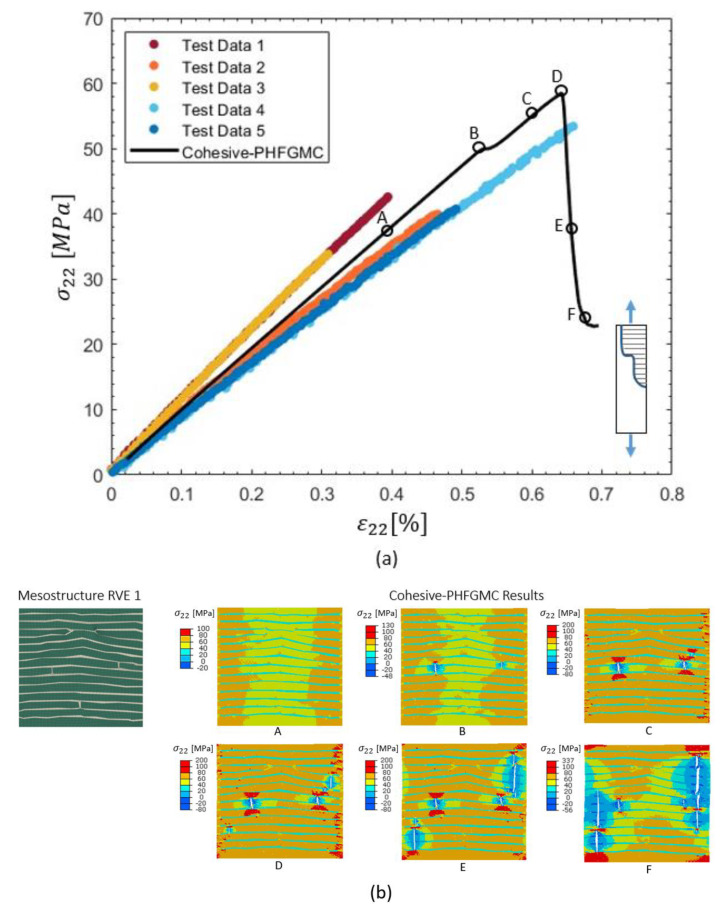
Cohesive PHFGMC analysis under transverse-tensile loading: (**a**) stress–strain response; (**b**) stress distribution and simulated crack propagation within the mesostructure RVE.

Transverse–tensile cohesive PHFGMC analysis results are presented in Figure 8. The top plot shows the transverse stress–strain response from five experimental tests and the cohesive PHFGMC prediction. Key points along the simulation curve (A–F) correspond to different stages of loading and crack evolution. The bottom row displays the corresponding transverse stress distributions within the RVE at these points, illustrating the progression of damage. Cracks first appeared in the matrix regions located within the tow-to-tow gaps and subsequently propagated into the tow regions. As loading continued, additional cracks initiated in the interlaminar resin-rich matrix areas and extended into adjacent tow domains, culminating in final failure. These results demonstrate the model’s capability to simulate damage initiation and growth patterns influenced by specific mesostructural features under transverse-tensile loading.

### 3.4. Parametric Study

**Figure 9:**
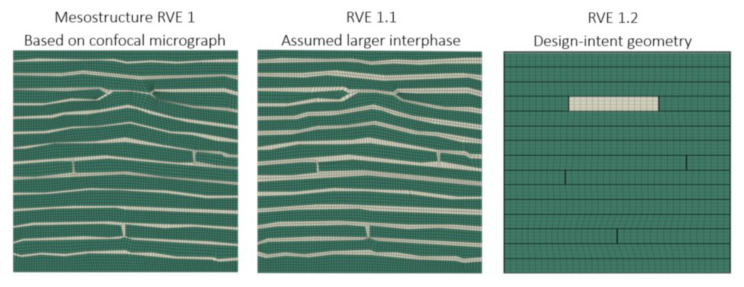
Three different PHFGMC-RVEs were employed for the parametric study: the mesostructured RVE 1 derived from the confocal micrograph; the second, RVE 1.1, developed from the same micrograph but with assumed extended interlaminar resin-rich areas; and the third, RVE 1.2, designed according to the manufacturing layout of the AFP process.

In this section, a parametric study is presented to examine the model’s sensitivity to assumed variations in mesostructure influenced by manufacturing parameters. The parametric study of laminate mesostructure RVEs is presented in Figure 9, featuring three varied PHFGMC-RVE configurations. The first RVE, “Mesostructure RVE 1”, is derived directly from a mesostructure micrograph, reflecting the observed geometry presented in the previous section. The second RVE, labeled “RVE 1.1,” is based on the same mesostructure micrograph but includes assumed extended interlaminar resin-rich regions. The third RVE, “RVE 1.2”, is based on the design-intent geometry of the AFP layup, representing the planned mesostructure configuration prior to any manufacturing-induced deviations.

The mesostructure RVE and the RVE 1.1 were derived from the same micrograph, sharing an identical gap pattern. However, RVE 1.1 featured assumed larger interlaminar resin-rich areas, increasing its volume fraction from 20% to 26%. To ensure consistency across all RVEs, the overall fiber volume fraction was maintained at approximately 60–61%. This was achieved by adjusting the internal fiber content within the tows according to their volume fraction within each RVE. In the mesostructure RVE (RVE 1), tows occupy 80% of the volume, while the remaining 20% consists of resin-rich regions located between the tows and at interlaminar interfaces. The measured fiber volume fraction within the tows is 76%, resulting in a total fiber content of approximately 60.8%.

RVE 1.1 was derived from the same micrograph as RVE 1 and retains the same tow gap pattern. However, to examine the influence of increased resin accumulation between plies, the interlaminar resin-rich regions were artificially extended, raising the total resin content to 26% and reducing the tow volume fraction to 74%. To preserve the same overall fiber volume fraction, the fiber content within the tows was increased to 82%. This parametric variation provides initial insight into how interlaminar resin content influences composite mechanical behavior and can serve as a foundation for further exploration of AFP manufacturing variability, particularly as studied in thermoplastic systems [20,28,52].

In contrast, RVE 1.2 represents the design-intent geometry of the AFP layup. It reflects the nominal gap placement and ply stacking as defined in the design, without manufacturing-induced deviations such as resin flow or fiber misalignment. RVE 1.2 includes a single resin gap measuring 0.8 mm in width and a thickness equal to the ply thickness. Unlike RVE 1 and RVE 1.1, RVE 1.2 excludes resin-rich interlaminar regions, which are considered byproducts of the manufacturing process and not part of the nominal design. As a result, the resin gap occupies approximately 10% of the RVE volume, and the remaining 90% consists of tows. To maintain the target overall fiber content, the fiber volume fraction within the tows was adjusted to 68%.

**Figure 10:**
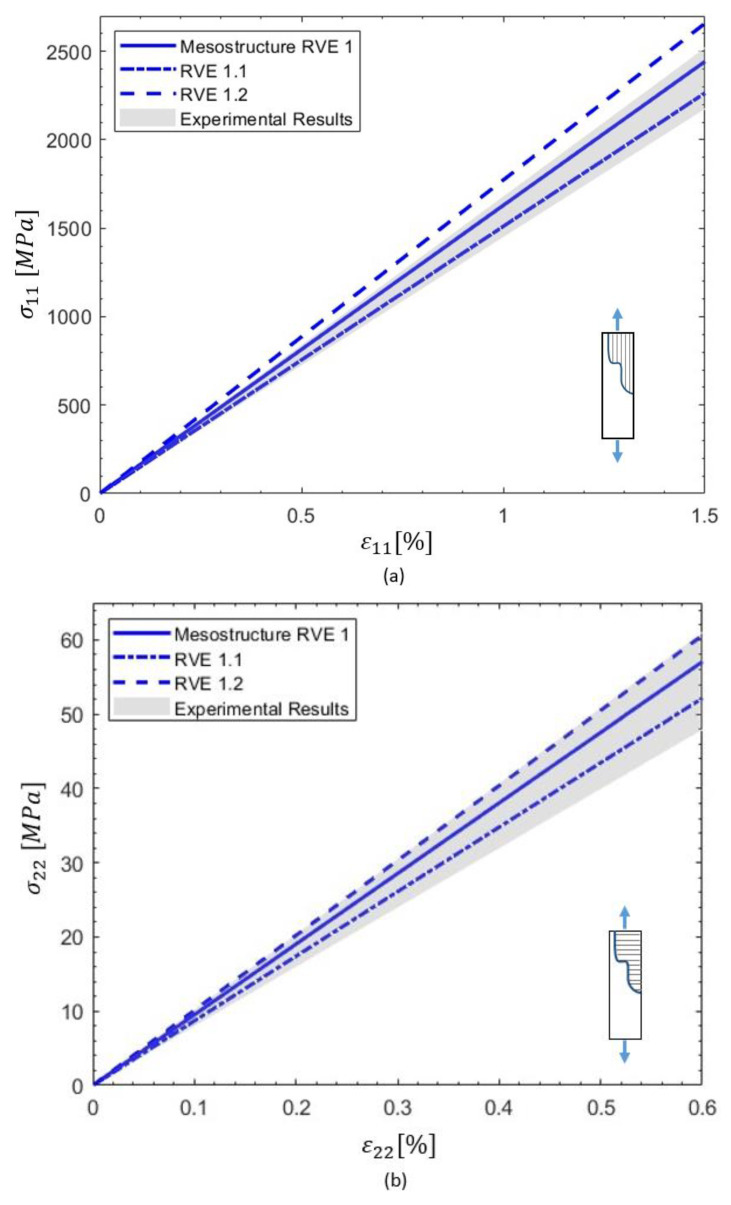

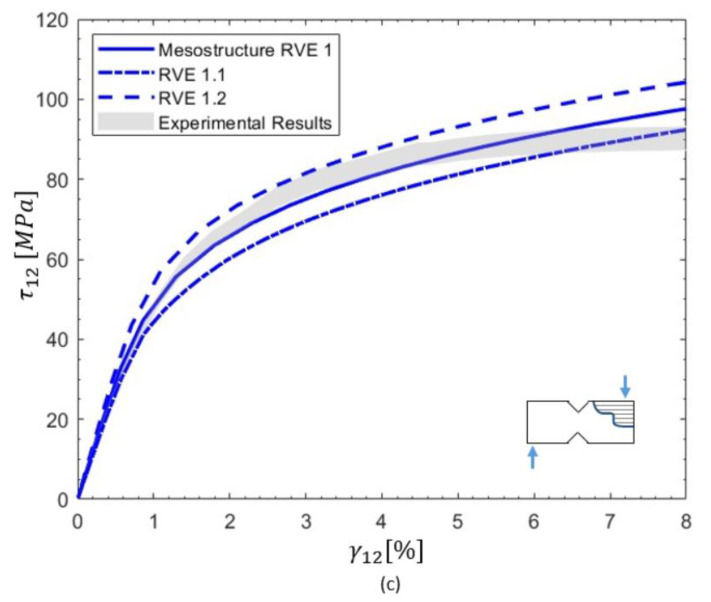
Predicted stress–strain responses from the parametric study of three RVEs under various loading conditions: (**a**) axial tension, (**b**) transverse tension, and (**c**) in-plane shear.

The tow properties for RVE 1.1 and RVE 1.2, as predicted by the PHFGMC using the microstructure RUC, are presented in Table 10. All other input parameters—including the tow’s nonlinear behavior and the linear and nonlinear properties of the M21 epoxy matrix (as shown in Table 6 and Table 7, respectively)—were kept consistent with those used in the mesostructure RVE. The PHFGMC-predicted stress–strain responses under axial tension, transverse tension, and in-plane shear loading are shown in Figure 10. A summary of the effective properties predicted for the mesostructured RVE, the extended interlaminar RVE 1.1, and the design-intent RVE 1.2 is provided in Table 11.

The relative errors between RVE 1 and RVE 1.1 were 3.7% in axial tension, 1.6% in transverse tension, and 5.3% in shear, suggesting that the mesostructure primarily influences the linear elastic behavior of longitudinal tension. Meanwhile, the transverse and shear responses are moderately affected by the increased interlaminar resin content, leading to stiffness reduction. The maximum relative errors between RVE 1 and RVE 1.2 were 10% in axial tension, 4.4% in transverse tension, and 6.6% in shear.

A comparison between the design-intent RVE and the mesostructure RVE revealed slightly higher theoretical axial and transverse tensile moduli for the design-intent case, as compared to both the mesostructure RVE and experimental results. The axial modulus predicted for RVE 1.2 was 180 GPa, closely matching the 178 GPa reported in the datasheet [39].

**Figure 11:**
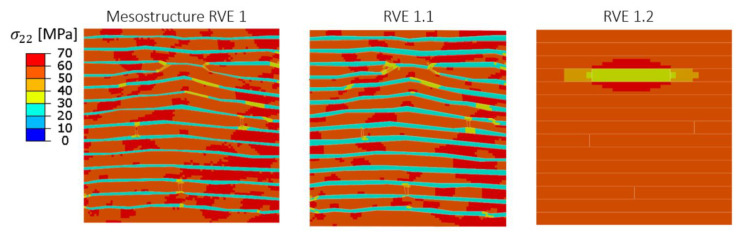
Local transverse tensile stress contours predicted by PHFGMC for different RVEs.

The transverse tensile stress contours predicted by the PHFGMC are shown in Figure 11. The results reveal differences in stress concentration between the mesostructure RVE and RVE 1.1, where the extended interlaminar resin regions influenced the stress distribution according to the gap pattern. In contrast, RVE 1.2 exhibited a distinctly different pattern, characterized by uniform stress within the tows and pronounced concentration near the resin gap. In this region, the stress in the epoxy significantly exceeded the levels observed in the interlaminar resin of RVE 1 and RVE 1.1, with the elevated stress extending into the adjacent tows. This comparison highlights the capability of the nested PHFGMC micro-to-meso framework to capture localized stress concentrations within AFP composites, providing accurate predictions of global mechanical response and internal stress distribution.

## 4. Conclusions

This study successfully developed and validated a hierarchically nested micromechanical modeling framework, the PHFGMC-AFP, for predicting the nonlinear mechanical behavior of AFP-manufactured carbon/epoxy composites. By explicitly incorporating key mesostructural features introduced during the AFP process—such as tow gaps and resin-rich regions—the two-level approach integrates micro-scale fiber/matrix interactions with meso-scale AFP-specific features derived from micrographs. The framework demonstrates excellent predictive accuracy with errors of 3.2% for longitudinal modulus and 1.3% for transverse modulus when validated against experimental results.

The investigation of multiple RVE configurations revealed that AFP composites exhibit structural robustness despite manufacturing variability, with minimal sensitivity to different gap patterns. The cohesive extension of the PHFGMC framework successfully captured damage initiation and crack propagation under transverse tensile loading, demonstrating that failure mechanisms initiate in gap regions before propagating into tow domains. The parametric study confirmed the model’s ability to distinguish between design-intent and as-manufactured responses, providing quantitative guidance for process optimization.

The PHFGMC-AFP framework offers computational efficiency while maintaining high fidelity, making it suitable for industrial applications including structural design optimization, quality control, and damage tolerance assessment. Overall, these findings highlight the structural robustness of AFP composites and demonstrate the proposed framework’s effectiveness as a tool for design evaluation. This work establishes a foundation for future research in progressive damage modeling, extension to additional composite systems, and integration into multiscale simulations for AFP composite design.

## Figures and Tables

**Table 1:** Dimensional characteristics of the specimens.

	Length [mm]	Width [mm]	Gage Length [mm]
Tensile specimens	140	12.7	40.0
Compression specimens	140	12.7	12.7
Shear specimens	76.0	20.0 (12.0 between the notches)	12.7

**Table 2:** Mechanical properties obtained from experimental results.

	ASTM Standard	Average Value	Standard Deviation
Longitudinal tensile modulus $E_{11,T}$[G Pa]	D3039	155.6	4.7%
Poisson’s ratio $\nu_{12}$	D3039	0.320	2.7%
Longitudinal compressive modulus $E_{11,C}$[G Pa]	D6641	142.2	4.1%
Transverse tensile modulus $E_{22,T}$[G Pa]	D3039	9.068	12%
Transverse compressive modulus $E_{22,C}$[G Pa]	D6641	9.420	18%
In-plane shear modulus $G_{12}$[G Pa]	D5379	5.411	3.3%

**Table 3:** Mechanical properties of IMA carbon fiber.

$E_{11}\left[\mathrm{GPa}\right]$	$E_{22}\left[\mathrm{GPa}\right]$	$E_{33}\left[\mathrm{GPa}\right]$	$\nu_{12}$	$\nu_{13}$	$\nu_{23}$	$G_{12}\left[\mathrm{GPa}\right]$	$G_{13}\left[\mathrm{GPa}\right]$	$G_{23}\left[\mathrm{GPa}\right]$
290	16.0	16.0	0.35	0.35	0.35	12.0	12.0	6.50

**Table 4:** Mechanical properties of M21 epoxy matrix.

E[GPa]	ν
4.30	0.32

**Table 5:** Effective properties of the tow predicted by the PHFGMC for the unidirectional hexagonal-based RUC.

$E_{11}\left[\mathrm{GPa}\right]$	$E_{22}\left[\mathrm{GPa}\right]$	$E_{33}\left[\mathrm{GPa}\right]$	$\nu_{12}$	$\nu_{13}$	$\nu_{23}$	$G_{12}\left[\mathrm{GPa}\right]$	$G_{13}\left[\mathrm{GPa}\right]$	$G_{23}\left[\mathrm{GPa}\right]$
200	10.4	10.4	0.34	0.34	0.38	7.42	7.42	3.95

**Table 6:** RO constants for nonlinear tow modeling.

$\sigma_{0}\left[\mathrm{MPa}\right]$	α	n
33.0	0.8	4.7

**Table 7:** Mechanical properties of M21 epoxy matrix, including RO constants for nonlinear modeling.

E[GPa]	ν	$\sigma_{0}\left[\mathrm{MPa}\right]$	α	n
4.30	0.32	11.5	0.0005	4.50

**Table 8:** Comparison of PHFGMC-Predicted and Experimental Elastic Properties for M21/34%/UD194/IMA-12K AFP Composites.

	$E_{11}\left[\mathrm{GPa}\right]$	$E_{22}\left[\mathrm{GPa}\right]$	$E_{33}\left[\mathrm{GPa}\right]$	$\nu_{12}$	$\nu_{13}$	$\nu_{23}$	$G_{12}\left[\mathrm{GPa}\right]$	$G_{13}\left[\mathrm{GPa}\right]$	$G_{23}\left[\mathrm{GPa}\right]$
PHFGMC	161	9.18	9.18	0.33	0.33	0.38	5.92	5.92	3.10
Experimental results	156 (Avg.) 165 (Max.)	9.07	-	0.32	-	-	5.41	-	-

**Table 9:** Cohesive parameters for matrix and tow subcells, defined according to the PPR traction–separation model [51].

	$\sigma_{\max}\left[\mathrm{MPa}\right]$	$\phi_{n}\left[\frac{N}{m}\right]$	α	$\lambda_{n}$	$\tau_{\max}\left[\mathrm{MPa}\right]$	$\phi_{t}\left[\frac{N}{m}\right]$	β	$\lambda_{t}$
Matrix regions	60	0.25	3	0.1	90	0.91	3	0.1
Tow regions	80	0.25	3	0.1	110	0.91	3	0.1

**Table 10:** PHFGMC-predicted tow properties used as inputs for RVE 1.1 and RVE 1.2.

	$E_{11}\left[\mathrm{GPa}\right]$	$E_{22}\left[\mathrm{GPa}\right]$	$E_{33}\left[\mathrm{GPa}\right]$	$\nu_{12}$	$\nu_{13}$	$\nu_{23}$	$G_{12}\left[\mathrm{GPa}\right]$	$G_{13}\left[\mathrm{GPa}\right]$	$G_{23}\left[\mathrm{GPa}\right]$
RVE 1.1 tow	225	11.3	11.3	0.34	0.34	0.38	7.40	7.40	4.38
RVE 1.2 tow	186	9.81	9.81	0.34	0.34	0.38	6.20	6.20	3.76

**Table 11:** Predicted effective elastic properties of different RVE microstructures.

	$E_{11}\left[\mathrm{GPa}\right]$	$E_{22}\left[\mathrm{GPa}\right]$	$E_{33}\left[\mathrm{GPa}\right]$	$\nu_{12}$	$\nu_{13}$	$\nu_{23}$	$G_{12}\left[\mathrm{GPa}\right]$	$G_{13}\left[\mathrm{GPa}\right]$	$G_{23}\left[\mathrm{GPa}\right]$
RVE 1	163	9.24	9.24	0.33	0.33	0.38	5.93	5.93	3.11
RVE 1.1	157	9.27	9.27	0.33	0.33	0.38	5.62	5.62	3.06
RVE 1.2	180	9.62	9.62	0.33	0.33	0.37	6.58	6.58	3.4

## Data Availability

The original contributions presented in the study are included in the article, further inquiries can be directed to the corresponding author.
